# Comparison of Sensory Recovery between Random Pattern Flap and Axial Pattern Flap in Finger Defect Reconstruction

**DOI:** 10.1055/a-2521-2291

**Published:** 2025-05-15

**Authors:** Tien Duc Nguyen, Thanh Dinh Trinh, Thuong Van Pham

**Affiliations:** 1 Department of Cosmetic Plastic Surgery, Faculty of Medicine, University of Medicine and Pharmacy at Ho Chi Minh City, Ho Chi Minh City, Vietnam; 2 Department of Surgery, Haiphong University of Medicine and Pharmacy, Haiphong, Vietnam

**Keywords:** random pattern flap, axial pattern flap, sensory recovery, soft tissue defects

## Abstract

**Background** This study aimed to investigate the association between the use of different flaps, including random and axial pattern flaps, and sensory recovery following finger soft tissue reconstruction using local pedicle flaps.

**Methods** A longitudinal study was conducted on 115 patients with 130 finger soft tissue defects treated with local pedicle flaps between December 2016 and December 2020. Assessments were made at early postsurgery (119 flaps), 3 months postsurgery (110 soft tissue defects), and 6 months postsurgery (94 soft tissue defects). Sensory recovery outcomes were compared between soft tissue defects reconstructed using random and axial pattern flaps.

**Results** In the early postsurgery period, there was a significantly higher prevalence of a static sense of two-point discrimination (s2PD) ≤6 mm among fingers with random pattern flaps (96.2%) than among fingers with axial pattern flaps (64.5%). The probability of s2PD ≤6 mm at the donor and recipient sites with the direct flap was 75.5% and 25.5%, respectively, which was significantly higher than that with the reversed flap. After 6 months, there was a significant difference in sensory recovery compared to that at 3 months postsurgery but not between different flap types.

**Conclusion** Sensory recovery after reconstruction was observed with all flap types, and better sensory recovery can be achieved in a shorter time postsurgery using random pattern flaps.

## Introduction

Finger injuries are among the most common reasons for emergency department visits. 1 Hand surgery specialists have proposed various reconstructive options for treating finger soft tissue defects, aiming to restore the normal length of the finger, cover defects with fat tissue under the skin, regain motor function and sensation, prevent stiffness, and enable a quick recovery to normal activities. 1 2

**Fig. 1:**
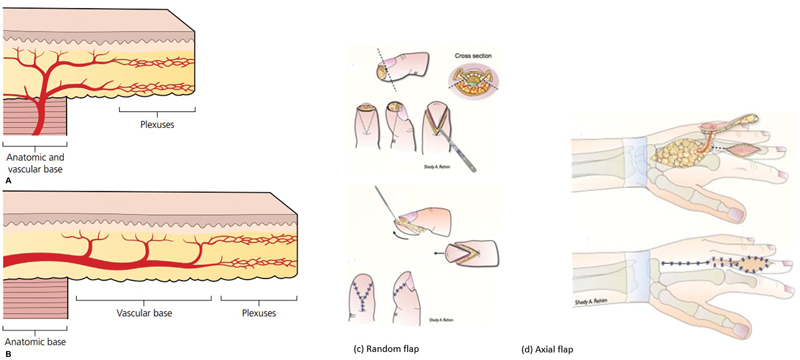
( **a** ) Illustration of (a) random and (b) axial flap reconstructions. ( **b** ) Illustrating design of (c) random- and (d) axial-flap on the hands.

When selecting a tissue flap for reconstructing finger soft tissue defects, key objectives include preserving tactile function, minimizing pedicle damage, and ensuring practicality and credibility postapplication. Options for reconstructing soft tissue defects in the fingers include local flaps, regional flaps, and distant flaps. Local flaps are preferred over distant flaps due to better tissue match and minimal morbidity, with improved sensory recovery compared to grafts. 3 Meanwhile, pedicle flaps are recommended for use on dorsal surfaces with good tissue mobility. 3 Random and axial pattern flaps are recognized as valuable local flaps for covering soft tissue defects. Based on their blood supply, these flaps are generally categorized as “random” and “axial” flaps ( Fig. 1a, b ). 4 Random pattern flaps rely on the subdermal vascular plexus, with a recommended length–width ratio not exceeding 3:1 for flap design. 5 Axial flaps received their blood supply from a named artery within the flap, with venous drainage primarily through corresponding veins ( Fig. 1b ). 3 6 While both types are frequently used in reconstructing finger soft tissue defects, there is limited evidence comparing their efficiency. Sensory recovery is a crucial indicator of the success of reconstruction. This study aimed to assess sensory recovery outcomes in finger soft tissue defect reconstruction using different types of flaps based on surgeons' experience.

## Methods

### Study Design and Patients

**Fig. 2:**
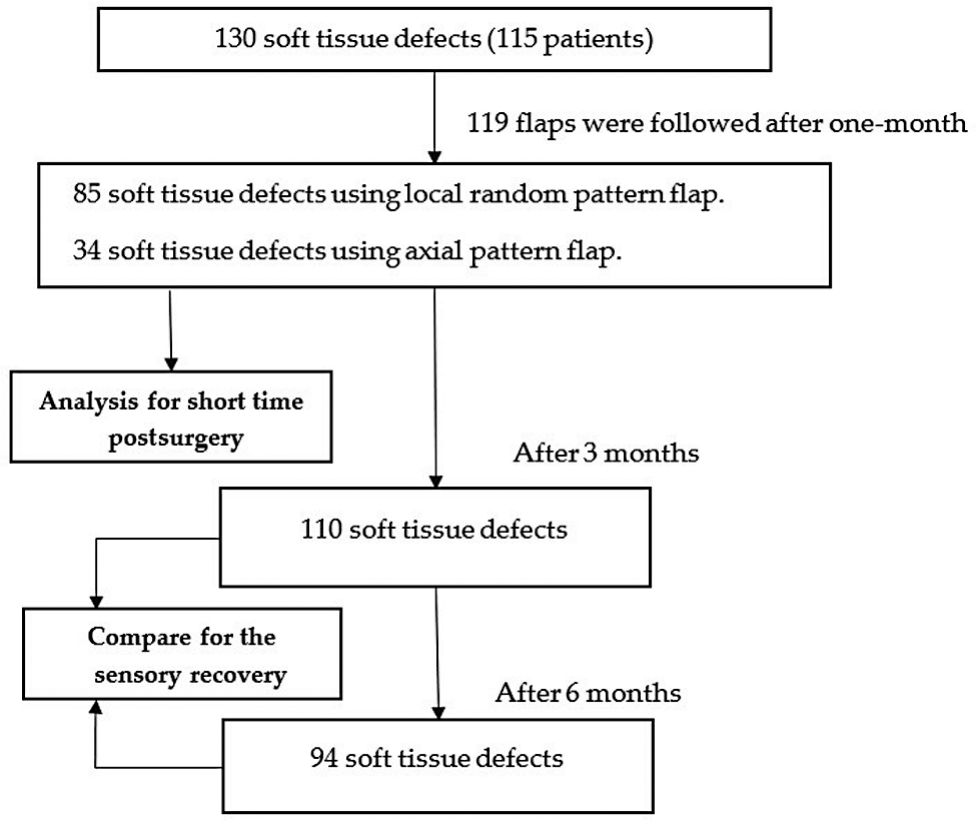
Study diagram.

A longitudinal study was conducted on 115 patients with 130 finger soft tissue defects who underwent reconstruction using a local pedicle flap from December 2016 to December 2020. Following surgery, the postsurgery outcomes of soft tissue defects were recorded at 1 month for 119 out of 130 fingers, at 3 months for 110 out of 119 fingers, and at 6 months for 94 out of 119 fingers ( Fig. 2 ). Written informed consent was obtained from all patients, and the study was approved by the Ethics Committee of Hanoi Medical University (approval number 215/HĐĐĐĐHYHN, dated December 2016).

Inclusion criteria for the study include patients with postinjury finger soft tissue defects resulting from various causes or postinfection debridement defects leading to the loss of subcutaneous fat tissue, exposing ligaments, and necessitating tissue flap coverage. Exclusion criteria included patients with life-threatening emergency surgical injuries requiring immediate attention, such as traumatic brain injury, multi-injury shock, penetrating chest trauma, or penetrating abdominal trauma. Patients with complete soft tissue defects on all fingers, late hospitalization with developing infections, or already undergoing other treatment methods like replantation, skin grafting, or stump bandaging were also excluded.

### Operative Procedures

#### Preparation

The wound was assessed to determine its orientation, whether horizontal or diagonal. Diagonal wounds were further evaluated by measuring the length of the soft tissue defects from the nearest to the farthest point along the finger. The length of the soft tissue defect was categorized into three groups: less than 11 mm, 11 to 20 mm, and over 20 mm.

#### Flap Types

The preferred order of flap selection was a local random pattern flap, followed by a direct axial pattern flap and a reversed axial pattern flap. The random-pattern flap was recommended for soft tissue defects requiring flap movement of less than 11 mm. The downstream axial pattern flap was utilized for defects requiring flap movement from 11 mm to 20 mm, while the inverted axial pattern flap was indicated for defects requiring flap movement exceeding 20 mm.

#### Techniques

The position of the axial and perforating vessels was determined using Doppler ultrasound to guide the design of the flap along the pedicle's path. After determining the blood supply, the flap was designed based on the lesion's morphology.

### Sensory Recovery Assessment

Sensory function was assessed using the Mackinnon–Dellon scale ( Table 1 ). 7 8 In this study, Grade 4 of the two-point discrimination test was used as the criterion for evaluating sensory recovery by categorizing the static sense of two-point discrimination (s2PD) into two groups: 6 and >6 mm.

**Table 1:** Mackinnon–Dellon scale of sensory recovery

Grade	Sensory recovery	s2PD (mm)	m2PD (mm)
S0	No recovery of sensibility in the autonomous zone of the nerve	–	–
S1	Recovery of deep cutaneous pain sensibility within the autonomous zone of the nerve	–	–
S1 ^+^	Recovery of the superficial pain sensibility	–	–
S2	Recovery of superficial pain and some touch sensibility	–	–
S2 ^+^	As in S2, but with an overresponse	–	–
S3	Recovery of pain and touch sensibility with the disappearance of overresponse	>15	>7
S3 ^+^	Same as S3 but the localization of the stimulus is good and there is an imperfect recovery of 2PD	7–15	4–7
S4	Complete recovery	2–6	2–3

Abbreviations: m2PD, motor sense of two-point discrimination; s2PD, static sense of two-point discrimination.

### Statistical Analysis

All analyses were conducted using Statistical Package for the Social Sciences version 20. Categorized variables were presented as numbers and percentages, while continuous variables were presented as means and standard deviations. The difference in prevalences was assessed using chi-square and Fisher's exact tests. The Wilcoxon signed-rank test was used to compare the means of s2PD and motor sense of two-point discrimination (m2PD) at 3 and 6 months postsurgery. A *p* -value of 0.05 was considered statistically significant.

## Results

In total, 115 patients with 130 soft tissue defects were included in this study. The characteristics of the enrolled patients are shown in Table 2 . Males accounted for 66.1% of the patients, with the main age group being 20 to 39 years old, comprising 54% of the total. The average size of the defects was 2.1 ± 3.1 cm ^2^ , with the areas of the random and axial pattern flaps measuring 1.5 ± 0.8 cm ^2^ and 3.7 ± 5.6 cm ^2^ , respectively. These differences were statistically significant ( *p* = 0.025). The dimensions of the tissue defects, including the length and width, were significantly smaller for random pattern flaps (1.3 ± 0.4 cm ^2^ and 1.2 ± 0.4 cm ^2^ ) than those of the tissue defects using axial pattern flaps (1.7 ± 0.1 cm ^2^ and 1.8 ± 1.6 cm ^2^ ; *p* < 0.05).

**Table 2:** General characteristics of participants according to the defects at baseline ( *n* = 130)

Characteristics		Total ( *n* , %)	Flap used		*p* -Value
			Random pattern flap ( *n* , %)	Axial pattern flap ( *n* , %)	
Gender	Male	86 (66.1)	61 (70.9)	25 (29.1)	0.532 a
	Female	44 (33.9)	34 (77.3)	10 (22.7)	
Age group	<20	17 (13.0)	13 (76.5)	4 (23.5)	0.775 a
	20–39	70 (54.0)	50 (71.4)	20 (28.6)	
	40–59	32 (24.3)	25 (78.1)	7 (21.9)	
	≥60	11 (8.7)	7 (63.6)	4 (36.4)	
Reason for hospitalization	Work accident	84 (64.3)	54 (64.3)	30 (35.7)	0.003 b
	Others	46 (35.7)	41 (89.1)	5 (10.9)	
Position of the finger	Right hand	59 (45.4)	47 (79.7)	12 (20.3)	0.165 a
	Left hand	71 (54.6)	48 (67.6)	23 (32.4)	
Direction of the wound	Horizon	77 (59.2)	70 (91.0)	7 (9.0)	<0.001 a
	Diagon	53 (40.8)	25 (47.2)	28 (52.8)	
Revealing tendons and bones at the wound		122 (93.8)	89 (93.7)	33 (94.3)	0.631 b
Wound dimension (cm)	Length (mean ± SD)	1.4 (0.6)	1.3 (0.4)	1.7 (0.1)	<0.001 c
	Width (mean ± SD)	1.3 (0.9)	1.2 (0.4)	1.8 (1.6)	0.036 c
Wound area	<2 cm ^2^	84 (64.3)	70 (83.3)	14 (16.7)	<0.001 a
	≥2 cm ^2^	46 (35.7)	25 (54.3)	21 (45.7)	
	Mean ± SD (cm)	2.1 (3.1)	1.5 (0.8)	3.7 (5.6)	0.025 c
Flap dimension (cm)	Length (mean ± SD)	2.0 (0.8)	1.9 (0.5)	2.5 (1.0)	<0.001 c
	Width (mean ± SD)	1.5 (1.1)	1.2 (0.4)	2.1 (1.8)	0.011 c
Flap area	Mean ± SD (cm)	3.2 (3.8)	2.3 (1.0)	5.8 (6.4)	0.003 c

Abbreviation: SD, standard deviation.

a Chi-square test.

b Fisher's exact test.

c Wilcoxon rank test.

Following surgery, 120 out of 130 soft tissue defect reconstructions were completely successful within the first week. Ten flaps experienced necrosis of less than one-third of the flap area ( Supplementary Table S1 [available in the online version only]). A total of 119 flaps were examined 1 month postsurgery.

The prevalence of s2PD less than or equal to 6 mm was 96.2% among random pattern flaps, significantly higher than 64.5% among the axial pattern flaps (odds ratio: 13.7; 95% confidence interval: 1.8–106.1; Table 3 ). At the donor sites, the percentage of s2PD less than or equal to 6 mm was 75.5% in direct flaps, and significantly higher than 28.5% in reversed flaps. In recipient sites, the percentage of s2PD 6 mm was 26.5%, with no instances recorded in the reversed flap group ( Table 4 ).

**Table 3:** The difference between flaps used and static sense of two-point discrimination 1 month postsurgery

s2PD (mm)	Random pattern flap ( *n* , %)	Axial pattern flap ( *n* , %)	OR (95% CI)	*p* -Value a
≤6	25 (29.4)	1 (2.9)	13.7	0.002
>6	60 (70.6)	33 (97.1)	(1.8–106.1)	

Abbreviations: CI, confidence interval; OR, odds ratio; s2PD, static sense of two-point discrimination.

a Fisher's exact test.

**Table 4:** The difference between the direction of flap usage and static sense of two-point discrimination 3 months postsurgery

s2PD		Direct flap ( *n* , %)	Reversed flap ( *n* , %)	OR (95% CI)	*p* -Value a
Donor sites (mm)	≤6	74 (75.5)	6 (28.5)	7.7	<0.001
	>6	24 (24.5)	15 (71.5)	(2.7–22.1)	
Recipient sites (mm)	≤6	26 (26.5)	0	–	0.007
	>6	72 (73.5)	21 (100)		

Abbreviations: CI, confidence interval; OR, odds ratio.

a Fisher's exact test.

**Fig. 3:**
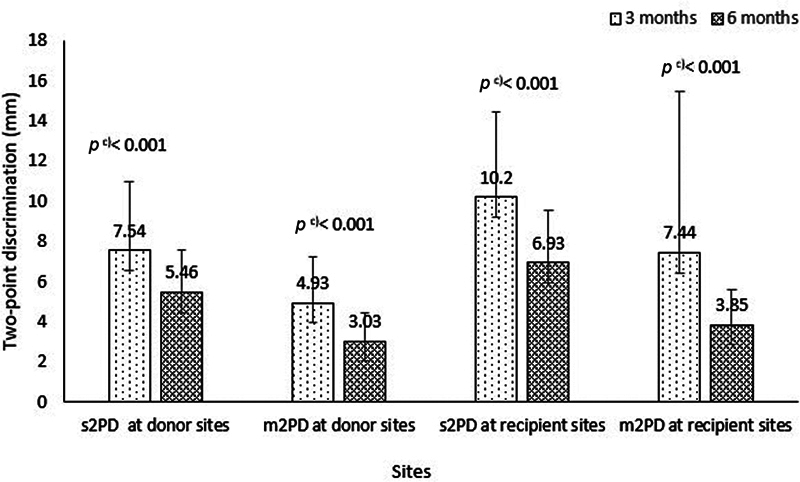
The difference of two-point discrimination 3 and 6 months postsurgery.

After 6 months postsurgery, a total of 94 soft tissue defect reconstructions were examined. There were no statistically significant differences in the prevalence of sensory recovery between soft tissue defect reconstructions using different flaps, including random or axial pattern flaps with direct or reversed flaps ( Supplementary Table S2 [available in the online version only]). The average s2PD at the donor and recipient sites was 5.46 ± 2.1 mm and 6.93 ± 2.6 mm, respectively. Additionally, the average m2PD was 3.03 ± 1.41 mm at the donor sites and 3.85 ± 1.77 mm at the recipient sites. It is worth noting that all two-point discrimination values significantly decreased compared to those measured after 3 months postsurgery ( Fig. 3 ).

**Fig. 4:**
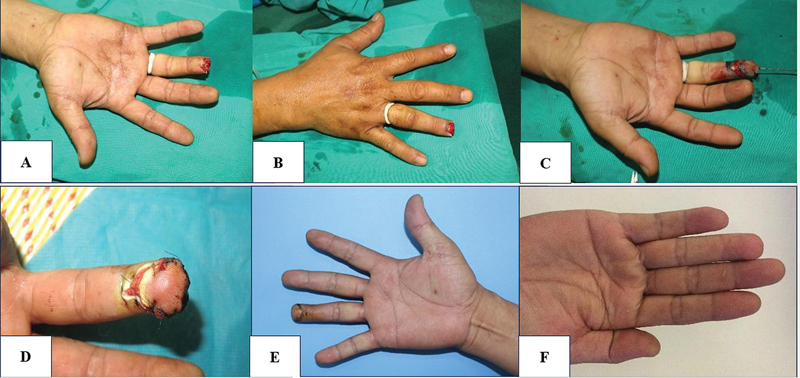
( **A** – **E** ) Preoperative, postoperative, and long-term follow-up photographs using an Atasoy flap (Case 1).

Case 1 ( Fig. 4 ): A 58-year-old man was hospitalized following an occupational accident that led to the amputation of the fourth finger of his right hand. The wound had jagged edges with bone exposure. We opt to reconstruct the area using an Atasoy flap. Six months later, full sensitivity was restored at both the donor and recipient sites.

**Fig. 5:**
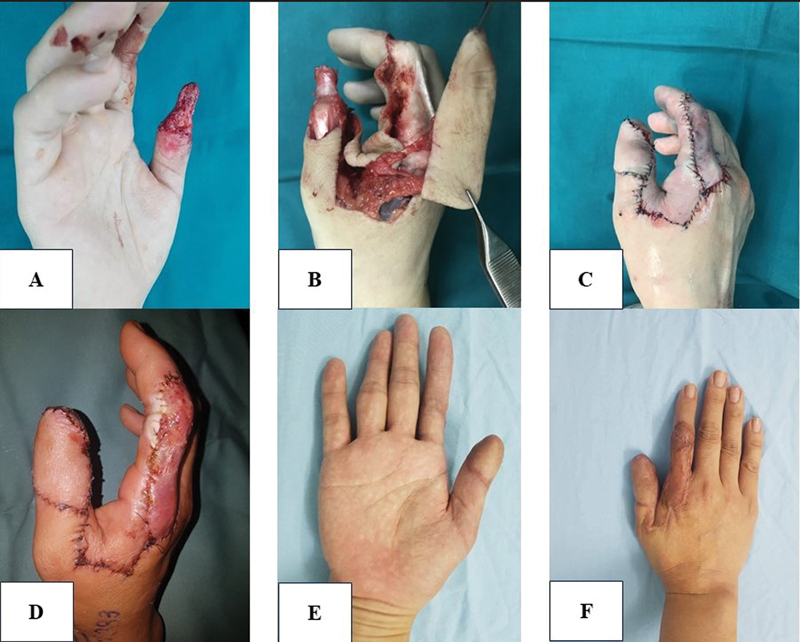
( **A–F** ) Preoperative, postoperative, and long-term follow-up photographs using an axial direct pattern flap (Case 2).

Case 2 ( Fig. 5 ): A 35-year-old woman underwent finger reconstruction for a soft tissue defect on the thumb of her right hand due to a press machine. An axial direct pattern flap was used to cover the defect. Six months later, the sensory function was completely recovered.

**Fig. 6:**
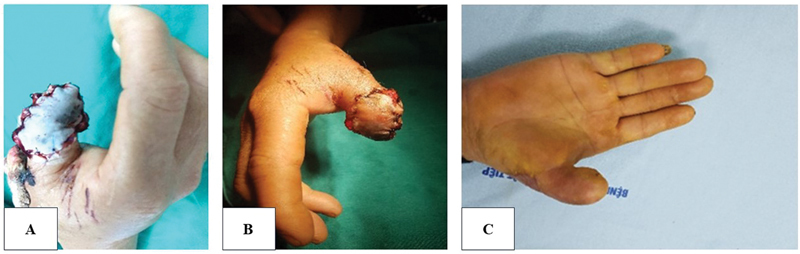
( **A–C** ) Preoperative, postoperative, and long-term follow-up photographs using an axial reverse-pattern flap (Case 3).

Case 3 ( Fig. 6 ): A 33-year-old woman presented with defects involving the distal and proximal phalanx of the thumb. A 4 × 2.5 cm axial pattern flap with a reversed direction was employed to cover the defect. Complete sensory recovery was achieved after 6 months.

## Discussion

Our findings indicate that there was good sensory recovery in fingers following soft tissue defect reconstruction using a local flap, with faster recovery observed in defects treated with a random pattern flap. However, long-term postsurgery assessments revealed sensory recovery in both random and axial pattern flaps.

The s2PD in fingers with random pattern flaps tended to be higher than in those with axial pattern flaps, suggesting a potentially better postsurgery recovery with random pattern flaps. Sungur et al's study demonstrated that all flaps could recognize axial pattern flaps within 6 mm 9 ; while research on 170 finger buds treated with local random pattern flaps showed a recovery in touch sensation after 3 months (range: 3–22 months; average: 9 months) with a two-point discrimination of 4 to 5 mm, averaging 4.5 mm. 10 These findings highlight the significant impact of local random pattern flaps on sensory recovery.

The flap's sensory recovery is influenced not only by the type of flap used, whether a random pattern or axial pattern, but also by the direction of flap movement, either direct or reversed. Direct flaps have shown higher rates of successful recovery compared to reversed flaps due to the anatomical arrangement of arteries and sensory nerves within the flaps. Arteries and sensory nerves that provide sensory and nutritional support to the fingers are aligned in a vascular bundle. While blood vessels in the flap allow bidirectional blood flow, sensory nerves only transmit signals in one direction. Therefore, direct flaps tend to recover more effectively than reversed flaps. Our findings are consistent with those of Chen et al 2014, 11 who reported two-point discrimination values of 8.3 mm for direct flaps and 10.4 mm for reversed flaps. Additionally, a study by Hirase et al in 1992 12 found excellent recovery of sensation with s2PD and m2PD values of 5 mm and 4.9 mm, respectively.

Our study findings demonstrate that all local flaps result in favorable postsurgery recovery outcomes. Two-point discrimination, in both motor and static states, showed progressive improvement over time. A previous study by Han et al 13 suggested that regardless of the flap type (direct or reversed) and nerve preservation, flaps typically recover within a year. There was no difference in recovery ability between sensing and nonsensing flaps, as reported by patients. Usami et al 2015 14 identified two key factors contributing to a successful recovery: adequate blood supply to the flaps and ensuring that the flap tissue closely resembles the lost tissue structure. This may explain why nerve dissection along the flap was deemed unnecessary, as it could pose technical challenges, prolong surgery time, and potentially affect flap survivability due to the risk of flap twisting. The hands and fingers have a rich blood supply, with superficial and deep palmar arches present in the palmar region. Additionally, two separate arteries supply blood to the fingers at the wrist. The blood vessels in the palm and wrist form a complex network with many branches connecting and penetrating each other. This intricate vascular system ensures that axial vessels supplying blood to the fingers are easily identifiable during dissection. Therefore, all finger flaps can be considered axial pattern flaps, with the dissection of the vascular pedicle being the determining factor. Besides, the researcher noticed that the flap's recovery ability depended on the thickness and flexibility of the covering tissue. Flaps sourced from the palm exhibited better and faster recovery in terms of touch sensation than those sourced from the wrist. Furthermore, complex grafts showed superior recovery outcomes compared to skin grafts.

This study still has several limitations. First, tissue loss, including factors such as defect size, site, depth, length, width, orientation, and composition, plays a crucial role in determining treatment outcomes. 3 Among them, the defect size is a key factor in selecting the appropriate flap and donor site. 15 16 Vizcay et al have clarified the different flaps for small reconstruction with an average size of 1.5 × 1.5 cm and for the large reconstruction with a mean dimension of 22 × 9.8 cm. 16 Koepple et al have suggested that the complication and duration of hospital stay might be attributed to the defect size. 17 Their study has shown variations in flap usage for different defect sizes, 17 highlighting the importance of considering defect size in recovery. However, this study did not assess the impact of these factors on defect recovery. Hence, the defect size bias should not be ruled out. Second, while changes in sensory function postoperation were documented, other demographic factors such as gender, age, hand dominance, smoking, drinking, and medical history may also influence sensory recovery in soft tissue defects.

### Conclusion

Long-term postsurgery follow-up revealed complete sensory recovery in all cases of soft tissue defect reconstruction using random or axial pattern flaps. However, better sensory recovery was observed in fingers reconstructed with random-pattern flaps during the early postsurgery period. Defect size, including the length and the width, was significantly different between groups using flap requiring confounder analysis. Further studies incorporating inferential statistics, defect size, and detailed patient characteristics, are needed to validate the efficacy of different flap types in restoring sensory function in soft tissue defect reconstruction.

## References

[1] Loréa P Chahidi N Marchesi S Ezzedine R Marin Braun F Dury M Reconstruction of fingertip defects with the neurovascular tranquilli-leali flap J Hand Surg [Br] 2006 31 03 280 284 10.1016/j.jhsb.2005.11.007 16403425

[2] Lee N H Pae W S Roh S G Oh K J Bae C S Yang K M Innervated cross-finger pulp flap for reconstruction of the fingertip Arch Plast Surg 2012 39 06 637 642 23233890 10.5999/aps.2012.39.6.637 PMC3518008

[3] Rehim S A Chung K C Local flaps of the hand Hand Clin 2014 30 02 137 151, v 24731606 10.1016/j.hcl.2013.12.004 PMC4011143

[4] Morrison E J Morrison W AJ Basic skin flaps and blood supply Plast Reconstr Surg 2015;(Mar):12 21

[5] Etzkorn J R Zito P M Hohman M H Council M Advancement flaps Coloproctology 2025 43 01 38 45 28613735

[6] Saber A Y Hohman M H Dreyer M A Basic Flap Design StatPearls Publishing 2024 33085399

[7] Ruijs A CJ Jaquet J B Kalmijn S Giele H Hovius S ER Median and ulnar nerve injuries: a meta-analysis of predictors of motor and sensory recovery after modern microsurgical nerve repair Plast Reconstr Surg 2005 116 02 484 494, discussion 495–496 16079678 10.1097/01.prs.0000172896.86594.07

[8] He B Zhu Z Zhu Q Factors predicting sensory and motor recovery after the repair of upper limb peripheral nerve injuries Neural Regen Res 2014 9 06 661 672 25206870 10.4103/1673-5374.130094 PMC4146230

[9] Sungur N Kankaya Y Yıldız K Dölen U C Koçer U Bilateral V-Y rotation advancement flap for fingertip amputations Hand (N Y) 2012 7 01 79 85 23448959 10.1007/s11552-011-9389-6 PMC3280365

[10] Aboulwafa A Emara S Versatility of homodigital islandized lateral V-Y flap for reconstruction of fingertips and amputation stumps Plast Reconstr Surg 2013 37 01 89 96

[11] Chen C Zhang W Tang P Direct and reversed dorsal digito-metacarpal flaps: a review of 24 cases Injury 2014 45 04 805 812 24315482 10.1016/j.injury.2013.11.002

[12] Hirasé Y Kojima T Matsuura S A versatile one-stage neurovascular flap for fingertip reconstruction: the dorsal middle phalangeal finger flap Plast Reconstr Surg 1992 90 06 1009 1015 1448495 10.1097/00006534-199212000-00012

[13] Han S K Lee B I Kim W K The reverse digital artery island flap: an update Plast Reconstr Surg 2004 113 06 1753 1755 15114141 10.1097/01.prs.0000117298.52225.43

[14] Usami S Kawahara S Yamaguchi Y Hirase T Homodigital artery flap reconstruction for fingertip amputation: a comparative study of the oblique triangular neurovascular advancement flap and the reverse digital artery island flap J Hand Surg Eur Vol 2015 40 03 291 297 24300507 10.1177/1753193413515134

[15] Kim S W Jung S N Sohn W I Kwon H Moon S H Ulnar artery perforator free flap for finger resurfacing Ann Plast Surg 2013 71 01 72 75 23503427 10.1097/SAP.0b013e31824681cc

[16] Vizcay M Pajardi G E Zanchetta F Stucchi S Baez A Troisi L Tailored skin flaps for hand reconstruction Plast Reconstr Surg Glob Open 2022 10 09 e4538 36203738 10.1097/GOX.0000000000004538 PMC9529032

[17] Koepple C Kallenberger A K Pollmann L Comparison of fasciocutaneous and muscle-based free flaps for soft tissue reconstruction of the upper extremity Plast Reconstr Surg Glob Open 2019 7 12 e2543 32537297 10.1097/GOX.0000000000002543 PMC7288888

