# Supplementary material for: Comparison of Sensory Recovery between Random Pattern Flap and Axial Pattern Flap in Finger Defect Reconstruction
Source: Arch Plast Surg. 2025 May 15;52(3):145–52. doi: 10.1055/a-2521-2291 (PMC12081082; doi:10.1055/a-2521-2291)
Supplement: Supplementary file 1 — Supplementary Material [file 10-1055-a-2521-2291-s23oct0478oa.pdf]

Supplementary Table S1 The results of reconstruction after a week (130 flaps)

Characteristics	Random pattern flap (*n*, %)	Axial pattern flap (*n*, %)	Total	*p*-Value[a]
Completely survive	94 (98.9)	26 (74.3)	120 (92.3)	0.002
Necrosis	1 (1.1)	9 (25.7)	10 (7.7)	

[a]Fisher's exact test.

Supplementary Table S2 The difference between the flaps used and static sense of two-point discrimination 6 months postsurgery (94 flaps)

s2PD (mm)	Random pattern flap (*n*, %)	Axial pattern flap (*n*, %)	*p*-Value[a]
≤ 6	62 (98.4)	30 (96.8)	1.0
>6	1 (1.6)	1 (3.2)	

Abbreviations: CI, confidence interval; OR, odds ratio; s2PD, static sense of two-point discrimination.
[a]Fisher's exact test.

 © 2025. The Author(s).
